# Ecological level analysis of primary lung tumors in dogs and cats and environmental radon activity

**DOI:** 10.1111/jvim.15936

**Published:** 2020-11-03

**Authors:** Brittany L. Fowler, Chad M. Johannes, Annette O'Connor, Deanna Collins, Jonathan Lustgarten, Chaohui Yuan, Kristen Weishaar, Kelly Sullivan, Kelly R. Hume, Jennifer Mahoney, Brittany Vale, Alicia Schubert, Valerie Ball, Katie Cooley‐Lock, Kaitlin M. Curran, Laura Nafe, Allison Gedney, Megan Weatherford, Dana N. LeVine

**Affiliations:** ^1^ Iowa State University College of Veterinary Medicine Ames Iowa USA; ^2^ Compassion First Pets Red Bank Veterinary Hospital Tinton Falls New Jersey USA; ^3^ Colorado State University Flint Animal Cancer Center Fort Collins Colorado USA; ^4^ Cornell University College of Veterinary Medicine Ithaca New York USA; ^5^ University of Pennsylvania Ryan Veterinary Hospital Philadelphia Pennsylvania USA; ^6^ Purdue Veterinary Teaching Hospital West Lafayette Indiana USA; ^7^ North Carolina State University College of Veterinary Medicine Raleigh North Carolina USA; ^8^ Mississippi State University College of Veterinary Medicine Mississippi State Mississippi USA; ^9^ Oregon State University Carlson College of Veterinary Medicine Corvallis Oregon USA; ^10^ Center for Veterinary Health Sciences Oklahoma State University Stillwater Oklahoma USA; ^11^ University of Georgia Veterinary Teaching Hospital Athens Georgia USA; ^12^ Present address: Brittany L. Fowler, Port City Veterinary Referral Hospital Portsmouth NH USA; ^13^ Present address: Annette O'Connor, Department of Large Animal Clinical Sciences, College of Veterinary Medicine Michigan State University East Lansing MI USA; ^14^ Present address: Jonathan Lustgarten, VCA, Inc. Los Angeles CA USA; ^15^ Present address: Kelly Sullivan, Guardian Veterinary Specialists Brewster NY USA; ^16^ Present address: Katie Cooley‐Lock, Charlotte Animal Referral & Emergency (CARE) Charlotte NC USA; ^17^ Present address: Laura Nafe, Department of Veterinary Medicine and Surgery University of Missouri College of Veterinary Medicine Columbia MO USA; ^18^ Present address: Allison Gedney, University of Pennsylvania Ryan Veterinary Hospital Philadelphia PA USA

**Keywords:** canine, epidemiology, feline, incidence, pulmonary neoplasia

## Abstract

**Background:**

Epidemiologic studies suggest residential radon exposure might increase the risk of primary lung cancer in people, but these studies are limited by subject mobility. This limitation might be overcome by evaluating the association in pets.

**Hypothesis:**

Primary pulmonary neoplasia (PPN) rate is higher in dogs and cats residing in counties with a high radon exposure risk (Environmental Protection Agency [EPA] zone 1) compared to zones 2 (moderate radon exposure risk) and 3 (low radon exposure risk).

**Animals:**

Six hundred ninety client‐owned dogs and 205 client‐owned cats with PPN.

**Methods:**

Retrospective review of medical records at 10 veterinary colleges identified dogs and cats diagnosed with PPN between 2010 and 2015. Each patient's radon exposure was determined by matching the patient's zip code with published county radon exposure risk. County level PPN rates were calculated using the average annual county cat and dog populations. The PPN counts per 100 000 dog/cat years at risk (PPN rates) were compared across radon zones for each species.

**Results:**

The PPN rate ratio in counties in high radon zone (1) was approximately 2‐fold higher than in counties in lower radon zones for dogs (rate ratio zone 1 to 2, 2.49; 95% confidence interval [CI], 1.56‐4.00; rate ratio zone 1 to 3, 2.29; 95% CI, 1.46‐3.59) and cats (rate ratio zone 1 to 2, 2.13; 95% CI, 0.95‐4.79; zone 1 to 3, 1.81; 95% CI, 0.9‐3.61).

**Conclusions and Clinical Importance:**

Exposure to household radon might play a role in development of PPN in dogs and cats.

**Abbreviations**
EPA: Environmental Protection Agency
ERL: environmental radon level (pCi/L)
PA: pulmonary adenocarcinoma
pCi/L: picocuries per liter
PPN: primary pulmonary neoplasia
USCBPS: United States Census Bureau population site

## 1 INTRODUCTION

According to the American Cancer Society, exposure to radon gas is thought to be the second leading cause of lung cancer in the United States.^1^ Radon‐222 is a colorless, odorless, and tasteless radioactive gas that is continually produced from the radioactive decay of uranium, which is found in all rocks and soil.^2^, ^3^ Radon has a short half‐life of 4 days, during which time the gas can move up through the ground to the outdoor air above and enter a home or other buildings through cracks and other penetrations in the structure's foundation.^2^ Radon gas decays into a series of solid radioactive particles that can be inhaled into the lung and deposited on the bronchial epithelium. Two of these decay products, polonium‐218 and polonium‐214, emit alpha particles that can cause DNA damage that could lead to lung cancer.^2^, ^3^

Lung cancer is the second‐most common cancer in people in the United States.^1^ One of every 14 men and 1 of every 17 women will develop primary lung cancer within their lifetime and lung cancer is the leading cause of cancer deaths among both women and men.^1^ Primary pulmonary neoplasia (PPN) is uncommon in dogs and cats, and little data are available regarding incidence rates, prognosis, treatment of choice, and benefits of adjunctive treatment. In addition, patients often are diagnosed late in the course of disease because they commonly are presented with nonspecific clinical signs (eg, lethargy, weight loss, coughing, vomiting), making effective treatment more difficult.^4^ Identifying a role for environmental radon in the pathogenesis of lung cancer in dogs and cats may lead to screening for and earlier diagnosis of PPN in pets residing in higher radon exposure areas.

There are several difficulties in establishing a causal association between radon exposure and lung cancer in people. Confounding factors include smoker status, exposure to secondhand smoke, and occupational hazard risk, all of which are difficult to control. Varied methods of radon dosimetry measurement, the mobility of people, and the inability to determine lifelong exposure to radon are confounding factors. People frequently will live in a variety of geographic regions with variable radon levels throughout their lifetime and they spend a substantial amount of time outside of their homes. Research on prolonged exposure to high radon within the environment started with cohort epidemiologic studies of underground miners in the 1970s, which consistently showed a strong dose‐response relationship between radon concentration to which miners were exposed and incidence of primary lung tumors. Numerous studies evaluating the relationship between radon exposure and lung cancer development have since been conducted, generating conflicting results.^5^, ^6^, ^7^, ^8^, ^9^, ^10^, ^11^, ^12^, ^13^ For example, a 1996 case‐control study of lung cancer among nonsmoking women in Missouri failed to show increased risk for study subjects exposed to domestic radon concentrations.^7^ In contrast, a population‐based case‐control study performed in Iowa in 2001 focusing on nonsmoking women who had lived in the same house for >20 years found a positive association between cumulative radon gas exposure and lung cancer.^8^ Many of the factors that make establishment of a causal relationship difficult in people may be partially ameliorated by studying pets. Dogs and cats tend to live the majority of their lives within the same house located within the same geographic radon zone, spend the majority of their day within the house, and do not have occupations that would increase their risk of radon exposure. By evaluating dogs and cats, we can perform a lifetime study knowing the patients were likely to be located within the same radon zone throughout their lives. It is important to understand whether lifetime radon exposure in cats and dogs is a predisposing factor to the development of lung cancer not only as a potential model for cancer in humans, but also to understand lung cancer pathogenesis and prevention in our pets. Given the paucity of information on the role of radon in companion animal cancer development, we aimed to determine the PPN incidence in dogs and cats in relationship to environmental radon levels (ERLs). We hypothesized that the incidence of PPN in dogs and cats would be higher in counties with higher environmental radon exposure risk.

## 2 MATERIALS AND METHODS

### 2.1 Study design: An ecological study with comparison of disease rates at the county level with county radon levels

Using the United States Environmental Protection Agency (EPA) map of county ERL, we determined the radon exposure risk of the county where each United States veterinary college is located.^14^ The EPA definition of radon zones is as follows: zone 1: counties with predicted average indoor radon screening >4 picocuries per liter (pCi/L); zone 2: counties with predicted average indoor radon screening from 2 to 4 pCi/L; and zone 3: counties with predicted average indoor radon screening <2 pCi/L.^14^

We contacted 8 veterinary colleges (2 from radon zone 1, 2 from zone 2, and 4 from zone 3) and asked them to participate in data collection and designate a contact person. The study proposal, design, and data collection methods were reviewed with a relevant person within the veterinary college (oncology service, internal medicine service, or medical records personnel). In addition to the veterinary colleges identified as described above, we searched the nationwide veterinary coding database Veterinary Medical Databases (VMDB) SNOWMED program for additional veterinary colleges that submitted data regularly to SNOWMED.^15^ We then searched the SNOWMED database for cases from the institutions that met our inclusion criteria (defined below). Once cases were identified, a co‐author at each site confirmed by local medical record review that inclusion criteria were met and obtained any missing data.

### 2.2 Case eligibility and identification

The veterinary medical records of the veterinary colleges (see Tables 1 and 2) and the SNOWMED database were searched to identify dogs and cats with PPN diagnosed between January 1, 2010, and December 31, 2015. Cases for inclusion were found by using the following search terms: “lung or pulmonary neoplasia,” “primary pulmonary mass,” and “pulmonary or lung tumor” within the internal medicine, oncology, surgery, and necropsy sections of the medical record systems.

**TABLE 1:** Participating veterinary colleges, their respective county Environmental Protection Agency (EPA) ranking of environmental radon level (ERL) and the residential radon zones of the dogs presenting to each respective veterinary college

		Cases per college per zone		
EPA radon zone	Veterinary college	ERL >4 pCi/L (zone 1)	ERL 2‐4 pCi/L (zone 2)	ERL <2 pCi/L (zone 3)
1	Colorado State University	186	11	5
	Cornell University	62	18	2
	Iowa State University	124	0	0
	Purdue University	47	19	1
2	North Carolina State University	1	7	29
	University of Georgia	2	15	3
3	Mississippi State University	2	18	25
	Oklahoma State University	1	2	18
	Oregon State University	0	3	24
	University of Pennsylvania	32	21	12

**TABLE 2:** Participating veterinary colleges, their respective county Environmental Protection Agency (EPA) ranking of environmental radon level (ERL) and the residential radon zones of the cats presenting to each respective veterinary college

		Cases per college per zone		
EPA radon zone	Veterinary college	ERL >4 pCi/L (zone 1)	ERL 2‐4 pCi/L (zone 2)	ERL <2 pCi/L (zone 3)
1	Colorado State University	42	1	0
	Cornell University	23	5	0
	Iowa State University	35	0	1
	Purdue University	14	5	0
2	North Carolina State University	2	11	6
	University of Georgia	0	3	1
3	Mississippi State University	0	3	6
	Oklahoma State University	1	0	7
	Oregon State University	0	1	8
	University of Pennsylvania	11	7	12

Possible cases then were further evaluated to determine eligibility. The diagnosis of PPN was based on ≥1 of the following: (1) diagnostic imaging that included documentation of a pulmonary mass on thoracic radiographs or thoracic computed tomography (CT) scan, with abdominal imaging to rule out the mass as metastasis from another site, (2) cytology of a pulmonary mass combined with abdominal imaging to rule out the pulmonary mass as metastasis from another site, or (3) histopathology of a pulmonary mass obtained during surgery or necropsy. Many identified cases included multiple methods for diagnosis. Patients were not eligible if imaging determined that the pulmonary mass was metastatic or if additional imaging was not performed to confirm the mass was the primary site of neoplasia.

Data requested from the review of the medical records included: age, sex, breed, reproductive status, presenting complaint, method(s) of diagnosis of the primary lung tumor (thoracic radiographs, thoracic CT, thoracic ultrasound examination, cytology of pulmonary mass, abdominal radiographs, abdominal ultrasound examination, histopathology [surgical or at time of necropsy]), type of lung tumor, if the diagnosis was definitive (definitively diagnosed by cytology or histopathology) or suspect (pulmonary mass found with the exclusion of other primary masses without a cytologic or histopathologic diagnosis), and zip code where the patient resided. For patients that had multiple entries regarding treatments or diagnostic tests related to the pulmonary mass within the medical record system, signalment at the time of PPN diagnosis was utilized. For patients with several diagnostic tests performed over a series of hospital visits, all diagnostic tests performed were included.

### 2.3 Pulmonary neoplasia incidence per dog/cat years at risk determination

The radon exposure of the pet is related to the county radon exposure risk where the pet resides rather than the county of the veterinary hospital where the pet was seen. The ERL of the pet's county of residence therefore was utilized for analysis. The PPN rates were calculated using the estimated annual dog and cat populations of each county instead of hospital case numbers. Annual county cat and dog populations in the country were determined as follows: The United States Census Bureau Population website (USCBPS) was used to obtain raw data for the 2010 human population by county.^16^ The website provided an estimate generator for the population by year, which was used to obtain estimates for the human population by county for the years 2011, 2012, 2013, 2014, and 2015. To obtain the estimated number of households per county for each year of the study (2010‐2015), the county population was divided by 2.6, the USCBPS estimate of the number of people per household.^16^ We then utilized the following equations from the American Veterinary Medical Association (AVMA) website to translate the number of households per county per year into the number of dogs and cats per county per year: (1) number of dogs per county = 0.584 × number of households, and (2) number of cats per county = 0.638 × number of households.^17^

Because the incidence of PPN differed by year, we calculated the average annual dog or cat population weighted by the number of PPNs diagnosed in that species each year. An example of such a calculation is given in the supplemental methods (Table S1). We then defined the rate as the number of outcomes (PPN cases) divided by time units (cat or dog years at risk). The number of cats or dogs with PPN over the 6‐year period was used as the outcome. The pet population was assumed to be stable and dynamic^18^ and therefore the weighted annual population of dogs or cats in the census was multiplied by 6 to represent the number of dog years (or cat years) at risk.

### 2.4 Statistical analyses

We first assessed potential models for the data. We used a likelihood ratio test to compare the fit of the Poisson model against the negative binomial model to the data. The *P* value indicated that the negative binomial model was the better fit. The response variable for the negative binomial model was the count of PPN cases offset by the years at risk, and the explanatory variable of interest was the EPA zone. The results are reported as the estimated incidence of PPN per 100 000 cat or dog years at risk and 95% confidence interval [CI] and comparative rate ratio and corresponding 95% CI for all possible pairwise comparisons of EPA zones. This analysis was conducted separately for dogs and cats. The analysis was conducted separately for definitive PPN cases and definitive and suspected PPN cases. As a post hoc analysis, we identified that 1 county (Larimer County, Colorado) had unusually high levels of PPN (dogs = 72, cats = 25) and we therefore conducted a separate analysis without this county to determine if these data were a highly influential data point (ie, if the association would remain after these data were removed). The intention of this post hoc analysis was not to remove these data from the report but to illustrate the impact of these data on the interpretation of the results. Statistical analysis was performed using commercially available software (R Core Team [2017]. R: A language and environment for statistical computing. R Foundation for Statistical Computing, Vienna, Austria. URL http://www.R-project.org/).

Information regarding the presenting complaint by species and analysis of species risk for a given clinical sign is provided in the supplemental material (Table S2, Supplemental Materials).

## 3 RESULTS

### 3.1 Veterinary college participation

Eight veterinary colleges representing all 3 radon zones agreed to participate in the study (Tables 1 and 2). The SNOWMED search identified 2 additional veterinary colleges that submitted cases regularly to SNOWMED within the study time period: Colorado State University College of Veterinary Medicine and Biomedical Sciences and Purdue University College of Veterinary Medicine. Tables 1 and 2 list the 10 participating veterinary colleges, their respective EPA radon zone, and the number of dogs and cats that resided in each radon zone and that presented to that veterinary college, respectively.

### 3.2 Dog demographics and lung tumor type

Medical record review identified 690 dogs with PPN from veterinary colleges with high (ERL rank 1, n = 475), mid‐level (ERL rank 2, n = 57), or low (ERL rank 3, n = 158) radon risk exposure. There was an even distribution of males to females (349/690 and 341/690, respectively) for dogs with lung tumors. The median age of dogs was 11 years (range, 6‐14.9 years). Ninety‐three percent (639/690) of the lung tumor patients were spayed or neutered. Breed distribution of lung tumor dogs was as follows: mixed breed dogs were the predominant breed within the study representing 15.5% of the population (107/690) followed by Labrador Retrievers (11.1%; 77/690), Golden Retrievers (4.8%; 33/690), Boxers (3%; 21/690), and Bernese Mountain Dogs (3%; 21/690). Other breeds identified included miniature Schnauzer (n = 15), German Shepherd (n = 13), Cocker Spaniel (n = 12), Beagle (n = 12), and smaller numbers of 49 additional breeds.

Given the variables for inclusion in the study, a cytologic or histopathologic diagnosis of the primary lung tumor was not required. A specific diagnosis (cytologic, histologic, or both) was obtained in 62% (n = 431) of dogs. Of these 431 cases, 31.6% (n = 136) were pulmonary carcinomas, 23.7% (n = 102) were pulmonary adenocarcinomas (PAs), and 18.6% (n = 80) were bronchoalveolar carcinomas (Table 3). Many of the cases included in the study with a definitive diagnosis used multiple approved methods of diagnosis (eg, thoracic radiographs, cytology of the pulmonary mass followed by surgery with biopsy) whereas the suspect PPN cases typically utilized thoracic CT and abdominal ultrasound examinations without additional imaging or follow‐up care.

**TABLE 3:** Types of canine and feline primary pulmonary neoplasms (PPN) with definitive diagnoses and method of diagnosis. The total patient number is reflective of the unique patients with that tumor type. Given that multiple methods of diagnosis (cytology, surgical biopsy, necropsy, or some combination of these) were used in a given patient, the individual methods of diagnosis do not add up to this total

	DOG (n = 431)			CAT (n = 110)		
	Surgical biopsy	Cytology	Necropsy	Surgical biopsy	Cytology	Necropsy
Bronchoalveolar carcinoma	80 (18.6%)			18 (16.4%)		
	42	28	31	7	1	10
Bronchogenic carcinoma	16 (3.7%)			6 (5.5%)		
	1	15	1	0	6	0
Histiocytic sarcoma	50 (11.6%)			1 (0.9%)		
	16	29	14	0	1	0
Pulmonary adenocarcinoma	102 (23.7%)			26 (23.6%)		
	69	44	28	6	8	16
Pulmonary carcinoma	136 (31.6%)			51 (46.4%)		
	33	94	29	11	38	14
Squamous cell carcinoma	11 (2.6%)			5 (4.5%)		
	7	4	3	0	2	3
Other	36 (7.9%)			3 (2.7%)		
	11	13	16	0	2	1

When evaluating the geographic distribution of cases, 254 counties from 35 states were represented. For the counties identified as having a dog with PPN, the number of cases per county over the study period ranged from 1 to 72 cases. The majority of all counties had 1 or 2 cases over the 6‐year period (median, 1). The counties with most cases were Larimer County, Colorado (n = 72) followed by Boulder County, Colorado (n = 30), and Polk County, Iowa (n = 23).

### 3.3 Cat demographics and lung tumor type

Medical record review identified 205 cats with PPN from veterinary colleges with high (ERL rank 1, n = 128), mid‐level (ERL rank 2, n = 36), or low (ERL rank 3, n = 41) radon exposure risk. There was an even distribution of male to female (105/205 and 100/205, respectively) cats with lung tumors in the study. The median age at tumor diagnosis was 12 years (range, 1‐20 years). Ninety‐eight percent of cats were spayed or neutered (200/205).

The most common breed represented within the study was the domestic shorthair accounting for 73.2% of the population (150/205), followed by the domestic longhair (11.7%; 24/205). There were 4 mixed breed cats (2%, 4/205) and 5 domestic medium hair (2.4%; 5/205). Several purebreds also were identified to have PPN, including Persian (2.9%, 6/205), Siamese (2.9%, 6/205), Himalayan (2%, 4/205), Russian blue (1%, 2/205), and 1 each of Burmese, Ragdoll, Abyssinian, and Norwegian forest cat (0.5%, 1/205).

Of the 205 cases identified, 110 had a specific diagnosis (cytologic, histologic, or both). Of those cases, 46.4% (n = 51) were pulmonary carcinomas, 23.6% (n = 26) were PAs, and 16.4% (n = 18) were bronchoalveolar carcinomas (Table 3).

When evaluating the geographic distribution of cases, 91 counties from 17 states were represented. Of the counties identified as having a cat with PPN, the number of cases per county over the 6‐year period ranged from 1 to 25 cases (median, 1). The counties with most cases were Larimer County, Colorado (n = 25) followed by Story County, Iowa (n = 12), Wake County, North Carolina (n = 11) and Philadelphia County, Pennsylvania (n = 11).

### 3.4 County radon zone and incidence of PPN in dogs

**FIGURE 1:**
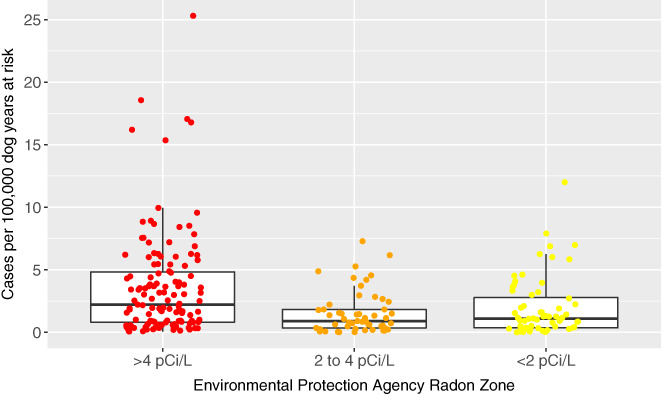
Box and whisker jittered plot representing the incidence of primary pulmonary neoplasia (PPN) per 100 000 dog years at risk by county in each radon level using cases with both suspect and definitive diagnoses. Each circular dot represents 1 county's neoplasia rate per 100 000 dog years at risk and is plotted based on the county's Environmental Protection Agency's (EPA) radon zone. The EPA definition of radon zones are as follows: zone 1: counties with predicted average indoor radon screening >4 pCi/L; zone 2: counties with predicted average indoor radon screening levels from 2 to 4 pCi/L; and zone 3: counties with predicted average indoor radon screening levels <2 pCi/L.^14^ The horizontal lines of the box represent the 25, 50, and 75% quartiles of the estimates

**FIGURE 2:**
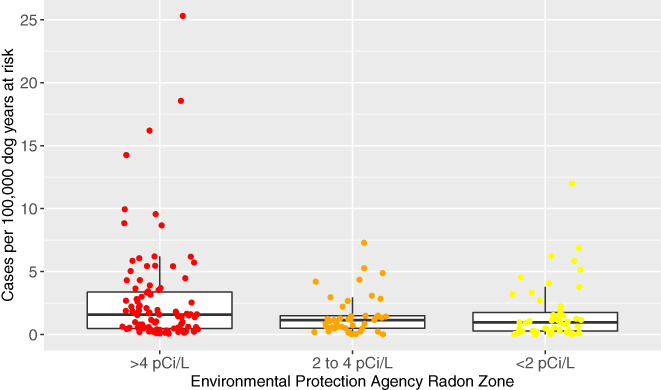
Box and whisker jittered plot representing the incidence of primary pulmonary neoplasia (PPN) per 100 000 dog years at risk by county in each radon level using only PPN diagnosed definitively. Each circular dot represents 1 county's neoplasia rate per 100 000 dog years at risk and is plotted based on the county's Environmental Protection Agency's (EPA) radon zone. The EPA definition of radon zones are as follows: zone 1: counties with predicted average indoor radon screening >4 pCi/L; zone 2: counties with predicted average indoor radon screening levels from 2 to 4 pCi/L; and zone 3: counties with predicted average indoor radon screening levels <2 pCi/L.^14^ The horizontal lines of the box represent the 25, 50, and 75% quartiles of the estimates

The incidence of PPN in dogs per 100 000 dog years at risk by county in each radon zone is represented graphically in Figure 1; a subanalysis with only dogs with definitive PPN diagnoses is included and represented in Figure 2. Larimer county had a very high PPN frequency compared to other counties, making it a possible outlier. Table 4 provides the mean PPN incidence in dogs per 100 000 dog years at risk in each radon zone. These data are presented including and excluding the Larimer County, Colorado, and for all dogs as well as those dogs with only definitive diagnoses.

**TABLE 4:** The estimated incidence of primary pulmonary neoplasia (PPN) for dog and cat populations with and without consideration of Larimer county Colorado per 100 000 dog or cat years at risk within each Environmental Protection Agency (EPA) radon exposure risk zone

	EPA designated radon zone	Estimated mean PPN incidence per 100 000 dog or cat years at risk	95% confidence interval
Definitive and suspect cases			
Dogs, all counties	1	2.66	(2.14‐3.32)
	2	1.07	(0.75‐1.54)
	3	1.2	(0.82‐1.65)
Dogs, Larimer, Colorado, excluded	1	2.46	(1.98‐3.08)
	2	1.06	(0.74‐1.52)
	3	1.15	(0.82‐1.62)
Cats, all counties	1	1.33	(0.96‐1.86)
	2	0.63	(0.34‐1.21)
	3	0.74	(0.44‐1.26)
Cats, Larimer, Colorado, excluded	1	1.17	(0.84‐1.65)
	2	0.62	(0.34‐1.17)
	3	0.72	(0.43‐1.21)
Definitive cases only			
Dogs, all counties	1	1.81	(1.4‐2.36)
	2	1.06	(0.71‐1.61)
	3	0.99	(0.67‐1.49)
Dogs, Larimer, Colorado, excluded	1	1.58	(1.22‐2.07)
	2	1.04	(0.70‐1.57)
	3	0.97	(0.66‐1.44)
Cats, all counties	1	0.97	(0.65‐1.44)
	2	0.39	(0.20‐0.81)
	3	0.53	(0.27‐1.01)
Cats, Larimer, Colorado, excluded	1	0.80	(0.53‐1.20)
	2	0.38	(0.20‐0.76)
	3	0.51	(0.27‐0.96)

Table 5 displays the PPN rate ratio in dogs among the different radon levels. The PPN incidence rate in counties with EPA radon zone 1 was approximately 2‐fold higher than that of counties with radon levels 2 or 3 (rate ratios, 2.49 and 2.29, respectively). However, the incidence rate in counties with radon level 2 does not appear to be different from radon level 3 as shown by the rate ratio being very close to the null value of 1 (ie, rate ratio, 0.92; 95% CI, 0.53‐1.59). For all estimates of rate ratios, a high level of uncertainty was reflected in the width of the CI of the rate ratios. When considering only dogs with definitive PPN diagnoses, the inferences remain the same (Tables 4, 5). The disease rate in counties with EPA radon zone 1 was approximately 2‐fold higher than those of counties with radon levels 2 or 3 (rate ratios, 1.71; 95% CI, 0.99‐2.95 and 1.82; 95% CI, 1.09‐3.06, respectively). However, the PPN incidence rate in counties with radon zone 2 does not appear to differ meaningfully from radon zone 3 as shown by the rate ratio being very close to the null value of 1 and the CI containing 1 (ie, rate ratio, 1.07; 95% CI, 0.0.57‐1.99). For all estimates of rate ratios, a high level of uncertainty was reflected in the width of the CI of the rate ratios.

**TABLE 5:** The rate ratio of primary pulmonary neoplasia (PPN) based on radon exposure. The ratio of disease rates for canine and feline PPN between Environmental Protection Agency (EPA) radon exposure risk zone (with and without consideration of Larimer county, Colorado). The rate ratios are expressed using definitive and suspect cases first and then with the definitive only cases

	EPA designated radon zone (numerator)	EPA designated radon zone (denominator)	Rate ratio (95% confidence interval)
Definitive and suspect cases			
Dogs, all counties	1	2	2.49 (1.56‐4.00)
	1	3	2.29 (1.46‐3.59)
	2	3	0.92 (0.53‐1.59)
Dogs, Larimer, Colorado, excluded	1	2	2.33 (1.46‐3.72)
	1	3	2.15 (1.38‐3.35)
	2	3	0.92 (0.54‐1.58)
Cats, all counties	1	2	2.13 (0.95‐4.79)
	1	3	1.81 (0.90‐3.61)
	2	3	0.85 (0.33‐2.15)
Cats, Larimer, Colorado, excluded	1	2	1.90 (0.86‐4.20)
	1	3	1.62 (0.82‐3.20)
	2	3	0.85 (0.34‐2.11)
Definitive cases only			
Dogs, all counties	1	2	1.71 (0.99‐2.95)
	1	3	1.82 (1.09‐3.06)
	2	3	1.07 (0.57‐1.99)
Dogs, Larimer, Colorado, excluded	1	2	1.52 (0.89‐2.58)
	1	3	1.63 (0.98‐2.71)
	2	3	1.08 (0.59‐1.98)
Cats, all counties	1	2	2.48 (0.99‐6.24)
	1	3	1.84 (0.78‐4.34)
	2	3	0.74 (0.25‐2.22)
Cats, Larimer, Colorado, excluded	1	2	2.09 (0.86‐5.10)
	1	3	1.56 (0.68‐3.60)
	2	3	0.75 (0.26‐2.14)

The estimates of mean incidence per 100 000 dog years at risk of PPN in dogs were lower in EPA radon zone 1 after removal of the Larimer County data (Table 4). Overall, the inferences were the same for both analyses (Table 5); an approximate 2‐fold increase in the rate of disease was observed in radon zone 1 counties compared to either radon zone 2 or 3 counties, with no evidence of substantially different rates of disease between radon county zones 2 and 3.

### 3.5 County radon zone and incidence of PPN in cats

**FIGURE 3:**
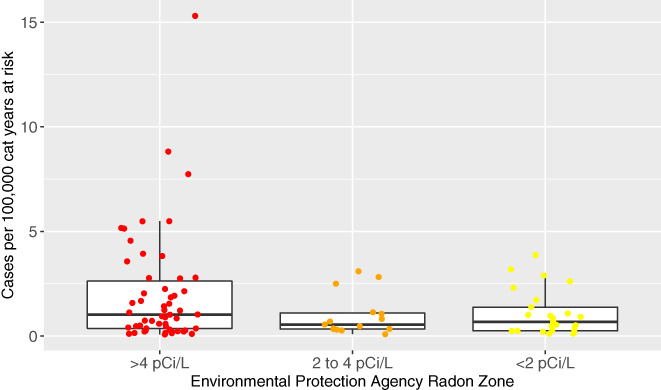
Box and whisker jittered plot representing the incidence of primary pulmonary neoplasia (PPN) per 100 000 cat years at risk by county in each radon level using cases with both suspect and definitive diagnoses. Each circular dot represents 1 county's neoplasia rate per 100 000 cat years at risk based and is plotted based on the county's Environmental Protection Agency's (EPA) radon zone. The EPA definition of radon zones are as follows: zone 1: counties with predicted average indoor radon screening >4 pCi/L; zone 2: counties with predicted average indoor radon screening levels from 2 to 4 pCi/L; and zone 3: counties with predicted average indoor radon screening levels <2 pCi/L. The horizontal lines of the box represent the 25, 50, and 75% quartiles of the estimates

**FIGURE 4:**
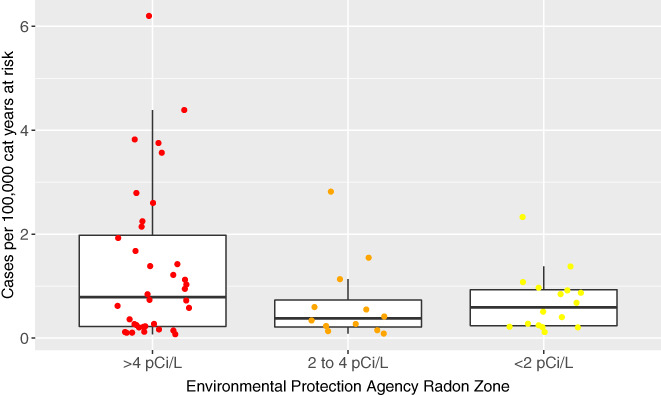
Box and whisker jittered plot representing the incidence of primary pulmonary neoplasia (PPN) per 100 000 cat years at risk by county in each radon level using only PPN diagnosed definitively. Each circular dot represents 1 county's neoplasia rate per 100 000 cat years at risk based and is plotted based on the county's Environmental Protection Agency's (EPA) radon zone. The EPA definition of radon zones are as follows: zone 1: counties with predicted average indoor radon screening >4 pCi/L; zone 2: counties with predicted average indoor radon screening levels from 2 to 4 pCi/L; and zone 3: counties with predicted average indoor radon screening levels <2 pCi/L. The horizontal lines of the box represent the 25, 50, and 75% quartiles of the estimates

The incidence of PPN in cats per 100 000 cat years at risk by county in each radon zone is presented graphically in Figure 3; a subanalysis with only cats with definitive PPN diagnoses is included, and is presented in Figure 4. Larimer county had a very high frequency compared to others, making it a possible outlier. Table 4 provides the mean PPN incidence in cats per 100 000 cat years at risk in the total population in each radon zone. These data are presented including and excluding Larimer County, Colorado, and for all cases in cats and for those cats with only definitive diagnoses.

Table 5 displays the PPN rate ratio in cats among 3 radon zones. The disease rate in counties with radon zone 1 was approximately 2‐fold higher than in counties with radon zone 2 (rate ratio, 2.13; 95% CI, 0.95‐4.79). The disease rate in counties with radon zone 1 was approximately 1.8‐fold higher than in counties with radon zone 3 (rate ratio, 1.81; 95% CI, 0.9‐3.61). The disease rate in radon zone 2 and radon zone 3 counties was similar, as indicated by a rate ratio very close to the null value of 1 (ie, rate ratio, 0.85; 95% CI, 0.33‐2.15). For all estimates of rate ratios, there is a higher level of uncertainty as compared with the data from dogs, reflected in the width of the CI of the rate ratios. When considering only cats with definitive PPN diagnoses, the inferences remained the same (Tables 4 and 5).

These data also were considered without the observation of the county Larimer, Colorado, given that there were 25 identified PPN cats in this county (Table 4). However, overall, the inferences were the same for both suspect and definitive cases combined, and definitive cases only (Table 5); an approximate 2‐fold increase in the rate of disease in radon zone 1 counties was observed compared to radon zone 2, an approximately 1.6‐fold increase in the rate of disease in radon zone 1 compared to radon zone 3, and no evidence of substantially different rates of disease between radon county zones 2 and 3.

## 4 DISCUSSION

Primary lung cancers are relatively rare in dogs and cats compared to humans.^19^ As such, very little is known about the pathogenesis of lung cancer in dogs and cats, and the effect environmental radon exposure may have on its development. It is crucial to determine the role that radon plays in the pathogenesis of lung cancer in companion animals to help advance preventative and treatment strategies for PPN. Defining the role of radon in dogs and cats also may provide information useful for human medicine, given that use of a companion animal population overcomes many of the confounding variables in radon studies of humans. Our retrospective systematic medical records review aimed to establish the county incidence of PPN per 100 000 animal years at risk and determine if PPN incidence rate was higher in counties with higher environmental radon exposure risk. We hypothesized that the incidence of PPN in dogs and cats would be higher in counties with higher environmental radon exposure risk.

We found that the incidence rate of PPN was higher in areas of higher ERLs for both dogs and cats. Unlike previous ecologic lung tumor studies of humans, a cat and dog study population offered a population of animals likely to have lived much of their lives in a single geographical location, and perhaps within the same household. These findings are hypothesis‐generating and indicate that higher ERLs might play a role in the development of PPN in dogs and cats.

The biologic foundation for radon's carcinogenic effects is well‐established. In 1994, the first study was performed that documented that malignant transformation of human bronchial epithelial cells can take place as a consequence of radon‐simulated alpha particles.^20^ Radiation can induce DNA damage, and the efficiency of the DNA‐damaging processes depends on the total dose, dose rate, and quality of the radiation.^21^ High linear energy transfer (LET) radiations, which include radon, are more cytotoxic and induce a higher rate of mutation per unit dose than do lower LET radiations, such as x rays and gamma rays.^21^ Radon‐induced DNA damage can cause cancer initiation, promotion, and progression by multiple mechanisms, including inducing chromosomal changes that persist in cellular progeny for generations and deleting tumor suppressor genes.^21^

Our results mirror what has been found in several studies of humans, including the 2001 epidemiologic study on Iowan women living in the same household for 20 years, which found a positive association between cumulative radon gas exposure and lung cancer.^8^ In addition, a hospital‐based case‐control study evaluated lung tumor patients from 2 hospitals in Spain and recorded histologic diagnosis, tobacco use, and residential radon measurement.^22^, ^23^ Radon exposure posed a risk even with low exposure for these patients, and exposure to tobacco smoke further increased the risk.^22^, ^23^ However, several large epidemiologic studies in people have not confirmed an association between radon exposure and primary lung tumor development.^6^, ^7^, ^9^ Speculated differences included errors in reconstructing past radon exposures,^7^ population mobility,^7^ small numbers of study subjects,^6^ inaccuracy of radon measurements,^6^ and occupational carcinogens.^6^ Dogs and cats offered a potentially superior model to humans for studying the association between ERL and incidence of lung cancer because companion animals tend to live in a single home the majority of their lives, often are housed mostly indoors, do not smoke, and do not have occupational carcinogen exposure. Furthermore, studies in humans struggle to reconstruct radon exposure from an early age whereas, given the shorter lifespans of dogs and cats, veterinary studies potentially can better capture entire lifetime radon exposure.

Our observational study found that the PPN rate in counties with radon zone 1 was approximately 2‐fold higher than that of counties with radon zones 2 or 3 for both dogs and cats. We also found that the rate of PPN tended to be lower in cats than dogs, but the comparative effect of the EPA zone was the same. Although the patterns are the same between dogs and cats, the estimates of rates in cats in each EPA zone and the rate ratios have wider CI, making conclusions less definitive. The smaller number of cats in our study compared to dogs likely accounts for some of this variation. The smaller number of cats may be a real difference or may simply be because of differences in veterinary care between the species. According to United States Pet Ownership statistics from 2012, although there were more cats per household (2.1 cats versus 1.6 dogs), dogs had more annual veterinary visits (2.6 visits per dog versus 1.6 per cats) and less money was spent on veterinary care for cats compared to dogs ($191 on cats per household per year versus $378 on dogs).^24^, ^25^ The pathogenesis of lung tumors also may differ in cats as compared with dogs, leading to a less clear contribution of radon exposure to PPN development in cats.

Although reports in the literature utilize various methods, making it challenging to directly compare rates among species, they also overall suggest a lower rate of PPN in cats compared to dogs. Previous studies in dogs include a large 2002 study evaluating neoplasia in dogs in the United Kingdom by evaluating a database of 130 684 insured dogs, which determined the age‐standardized incidence rate of lung cancer to be 15 cases per 100 000 dogs per year.^26^ In a closed colony of 398 beagle dogs, 35 dogs developed primary lung carcinoma over their lifetimes (8.8% cumulative incidence).^27^ Similar data for cats are lacking. To our knowledge, only 1 similar population study in cats was performed over 30 years ago, and found that PPN was diagnosed in 2.2 of 100 000 cats.^28^ More difficult to directly compare to population studies of dogs, a previous study evaluating pulmonary carcinomas in cats admitted to the Veterinary Teaching Hospital at North Carolina State University from 2006 to 2010 showed an incidence of 0.69% over that time period.^19^ In contrast, a pathology study described more PPN in cats (0.75% of all accessions for cats) compared to dogs (0.58% of all accessions for dogs).^29^ Although previous epidemiologic data in the veterinary literature is sparse, our data combined with most of the data available in the literature suggest a lower PPN rate in cats compared to dogs. As previously suggested, the fact that cats receive less veterinary care than dogs also may account for some of this difference. ^24^, ^25^

Genetic susceptibility also contributes to lung cancer development. Human medicine has focused substantial attention on genetic markers that increase the risk of PPN. The most relevant genes associated with lung cancer in humans include EGFR, KRAS, MET, LKB1, BRAF, PIK3CA, ALK, RET, and ROS1.^30^ We did not evaluate for genetic factors that may play a role in the development of PPN in dogs and cats. Future studies should explore the interplay of genetic alterations and radon exposure in the development of PPN.

One potential concern regarding our patient population is that all cases were extracted from referral hospitals. Because clients seeking specialty care may have more disposable income, they may have been more likely to have radon mitigation systems in their homes. If this were true, our patient population should have had the least likelihood to develop radon‐induced PPN, which would decrease the association between radon category and PPN. Given that this potential bias would shift the comparison of PPN rates toward the null hypothesis, our findings actually are more likely to be meaningful. Additionally, because our study evaluated cases diagnosed regardless of whether treatment was pursued, the socioeconomic status of the client would be less likely to have biased the patient population.

Our study also had several limitations because of its retrospective design. First, we were dependent on EPA radon zones, rather than individual household radon measurements. Radon levels can vary in a given region and even within a home.^31^ Record review did not allow us to determine where animals were housed (indoor, indoor and outdoor, outdoor). In addition, evaluation of the medical records did not allow for determination of whether or not the dogs and cats resided in households with smokers, which may be important because previous studies have found conflicting evidence regarding a potential association between secondhand smoke and increased risk of developing lung cancer in companion animals.^32^, ^33^ The environmental setting of the individual house also was undetermined in our study (urban versus suburban versus rural). Similarly, our record review did not allow us to determine how much of each animal's life was spent in 1 location or 1 household, how long they were exposed to each radon level and whether or not the exposure occurred previously in another home or shelter. Although some of our assumptions may have been incorrect, they would only interfere with the statistical analysis if they occurred differentially based on the EPA radon exposure zone, which is unlikely. Also, because we only analyzed patients that presented to veterinary colleges, our county PPN may be underestimated, but this is unlikely to have impacted the relationship between PPN rate and radon level. In addition, not all animals had a confirmed definitive diagnosis of PPN, and misdiagnosis was possible (which could lead to overestimating the rate of PPN). We attempted to exclude nonprimary lung tumors by determining that appropriate complete imaging of the chest and abdomen had been performed. The data were reanalyzed using only cases that were diagnosed definitively and the same inferences were made. Lastly, we ideally would have compared PPN rate to the rate of other neoplasms to ensure there was a unique association between PPN and radon versus cancer in general. We collected information on other cancer cases from all veterinary colleges, but discrepancies in data collection methods and lack of zip code information per case (given that we ultimately utilized county information by patient zip code) prohibited us from using this data.

Although our results are intriguing and suggest an association between PPN development and radon exposure in cats and dogs, interpretation must be made cautiously. Ours was a hypothesis‐generating study, and can be a pilot for future prospective studies to confirm our findings at the individual household level. Ideally, prospective studies would quantify radon exposure with in‐house radon dosimeters and would include only smoke‐free households. Such studies would be helpful to further elucidate the role of environmental radon in the development of lung tumors in dogs and cats, provide insight into the role of lifetime radon exposure in lung cancer development in humans, and ultimately inform development of targeted treatments or strategies to decrease environmental radon exposure.

## CONFLICT OF INTEREST DECLARATION

Authors declare no conflict of interest.

## OFF‐LABEL ANTIMICROBIAL DECLARATION

Authors declare no off‐label use of antimicrobials.

## INSTITUTIONAL ANIMAL CARE AND USE COMMITTEE (IACUC) OR OTHER APPROVAL DECLARATION

Authors declare no IACUC or other approval was needed.

## HUMAN ETHICS APPROVAL DECLARATION

Authors declare human ethics approval was not needed for this study.

## Supporting information


**Appendix** S1: Supporting Information.
