# Supplementary material for: Ecological level analysis of primary lung tumors in dogs and cats and environmental radon activity
Source: J Vet Intern Med. 2020 Nov 3;34(6):2660–70. doi: 10.1111/jvim.15936 (PMC7694848; doi:10.1111/jvim.15936)
Supplement: Supplementary file 1 — Appendix S1: Supporting Information. [file JVIM-34-2660-s001.pdf]

Supplemental Information

Supplemental Methods

Calculation of weighted average annual dog/cat populations

As the incidence of PPN differed by year, we calculated the average annual dog/cat population weighted by the number of PPNs diagnosed in that species each year. Using Boulder County Colorado (Table S1) as an example county, the number of dogs with PPN and the estimated annual county dog population are provided, and calculated as described in the main methods section. We calculated the weighted average annual canine population from 2010-2015 as follows: (2/30)*66164 + (3/30)*67519 + (6/30)*68586 + (5/30)*69695 + (12/30)*70350 + (2/30)*71593 = 69,409.

Table S1. Canine population and primary pulmonary neoplasia (PPN) occurrence in Boulder County Colorado. Data from this table were used in the methods to demonstrate calculation of a county canine population weighted by the number of PPN diagnosed that year.

Year	# Dogs	Estimated Dog Population
2010	2	66164
2011	3	67519
2012	6	68586
2013	5	69695
2014	12	70350
2015	2	71593

Assessment of variation in clinical presentation between cats and dogs

To determine if clinical signs differ by species, we calculated relative risk of cats, compared to dogs, presenting with the following symptoms: cough, respiratory distress, weight loss, lethargy, pleural effusion, vomiting, anorexia, or an incidental finding (Dean AG, Sullivan KM, Soe MM. OpenEpi: Open Source Epidemiologic Statistics for Public Health, Version. www.OpenEpi.com, updated 2013/04/06, accessed 2018/8/05).

Supplemental Results

Although unrelated to the influence of radon on PPN development, we documented canine and feline clinical signs and the relative risk of each sign depending on species due to the lack of this information in the veterinary literature.

Canine Presenting Complaint

As shown in Supplemental Table 2, the most common presenting complaint among dogs with primary lung tumors was coughing, seen in 40.6% dogs at presentation (280/690). When compared to cats, dogs were 2.4 times (CI 1.78 – 3.37) more likely to present to the hospital for coughing. Twelve percent (83/690) of the dogs presented in distress with tachypnea or dyspnea and 10.7% (74/690) presented with lethargy. 10.4% (72/690) of the dogs presented to their respective veterinary teaching hospital as a referral for an incidental pulmonary mass found on imaging with a lack of clinical signs.

Feline Presenting Complaint

When evaluating the presenting complaints among cats, no clear “common sign” was identified. Coughing was only seen at the time of presentation in 16.6% of patients (34/205). In contrast to the canine study population, a higher percentage of cats presented to the veterinary college in respiratory distress (15.6%, 32/205). Cats were 1.3 times (CI 0.89 – 1.89) more likely to present

with respiratory distress compared to dogs and 3.7 times (CI 1.6 – 8.59) more likely to present with pleural effusion. In addition, cats were 4.6 times (CI 2.8 – 7.49) more likely to present for evaluation due to weight loss than dogs, and 1.7 times (CI 1.13 – 2.52) more likely to present with anorexia (see Supplemental Table 2). 13.2% (27/205) of the cats presented to their respective veterinary teaching hospital as a referral for an incidental pulmonary mass found on imaging with a lack of clinical signs.

Table S2. The frequency count (percentage) of presenting complaints of 895 dogs and cats with primary pulmonary lung tumors. The relative risk was calculated to compare clinical signs between cats and dogs with 95% confidence intervals noted. The asterisk (*) indicates a risk ratio for which the confidence interval does not contain 1.

Presenting Complaint	Dogs (n = 690)	Cats (n = 205)	Relative Risk (95% confidence interval) for clinical signs (cats vs dogs)
**Anorexia/Hyporexia**	62 (9%)	31 (15.1%)	1.7 (1.13 – 2.52) *
**Coughing**	280 (40.6%)	34 (16.6%)	0.4 (0.30 – 0.56) *
**Respiratory distress**	83 (12%)	32 (15.6%)	1.3 (0.89 – 1.89)
**Lethargy**	74 (10.7%)	31(15.1%)	1.4 (0.96 – 2.08)
**Pleural Effusion**	10 (1.4%)	11 (5.4%)	3.7 (1.6 – 8.59) *
**Vomiting**	36 (5.2%)	19 (9.3%)	1.8 (1.04 – 3.03) *
**Weight Loss**	25 (3.6%)	34 (16.6%)	4.6 (2.8 – 7.49) *
**Incidental Finding**	72 (10.4%)	27 (13.2%)	1.6 (1.09 – 2.32) *
